# Developmentally Regulated Innate Immune NFκB Signaling Mediates IL-1α Expression in the Perinatal Murine Lung

**DOI:** 10.3389/fimmu.2019.01555

**Published:** 2019-07-10

**Authors:** Brittany Butler, Robyn De Dios, Leanna Nguyen, Sarah McKenna, Sankar Ghosh, Clyde J. Wright

**Affiliations:** ^1^Section of Neonatology, Department of Pediatrics, University of Colorado School of Medicine, Aurora, CO, United States; ^2^Department of Microbiology and Immunology, College of Physicians and Surgeons, Columbia University, New York, NY, United States

**Keywords:** rodent, endotoxin shock, inflammation, signal transduction, lung

## Abstract

Bronchopulmonary dysplasia (BPD) is the most common morbidity complicating premature birth. Importantly, preclinical models have demonstrated that IL-1 receptor antagonism prevents the lung injury and subsequent abnormal development that typically results following perinatal exposure to inflammatory stresses. This receptor is activated by two pro-inflammatory cytokines, IL-1α and IL-1β. While many studies have linked IL-1β to BPD development, IL-1α is relatively under-studied. The objective of our study was to determine whether systemic inflammatory stress induces IL-1α expression in the neonatal lung, and if so, whether this expression is mediated by innate immune NFκB signaling. We found that endotoxemia induced IL-1α expression during the saccular stage of neonatal lung development and was not present in the other neonatal organs or the adult lung. This IL-1α expression was dependent upon sustained pulmonary NFκB activation, which was specific to the neonatal lung. Using *in vivo* and *in vitro* approaches, we found that pharmacologic and genetic inhibition of NFκB signaling attenuated IL-1α expression. These findings demonstrate that innate immune regulation of IL-1α expression is developmentally regulated and occurs via an NFκB dependent mechanism. Importantly, the specific role of developmentally regulated pulmonary IL-1α expression remains unknown. Future studies must determine the effect of attenuating innate immune IL-1α expression in the developing lung before adopting broad IL-1 receptor antagonism as an approach to prevent neonatal lung injury.

## Introduction

Bronchopulmonary dysplasia (BPD) remains one of the most common morbidities associated with preterm birth, affecting 10-15,000 infants in the United States annually (1, 2). Exposure to inflammatory stress, including chorioamnionitis, early-onset, and late-onset sepsis increase the risk of developing BPD (3–6). Evidence of an active pro-inflammatory innate immune response can be measured in the amniotic fluid, serum, and tracheal aspirates of infants that go on to develop BPD (7–9). Despite these associations, the current paradigm maintains that early life immunity is marked by an impaired ability to mount a pro-inflammatory innate immune response (10–17). This contradiction highlights the gap in our understanding of the mechanisms linking the innate immune response to neonatal lung injury and leaves us without safe and effective anti-inflammatory therapies to prevent BPD in at risk infants (18, 19). A better understanding of the mechanisms and transcriptional selectivity of the neonatal innate immune response that explain these observations could lead to the identification of new therapeutic targets to prevent ongoing injury to developing organs.

Multiple laboratory studies indicate that pro-inflammatory IL-1 signaling plays a central role in mediating neonatal lung injury and abnormal development (20–22). Two distinct IL-1 genes produce proteins that bind the cell surface IL-1 receptor (IL1RI): IL-1α and IL-1β. The role of IL-1β in mediating neonatal lung injury has been the subject of intense study. Even in the absence of inflammatory stress, pulmonary overexpression of IL-1β during the saccular stage disrupts lung development (23, 24). Furthermore, neonatal treatment with IL-1 receptor antagonist attenuates hyperoxia-induced lung injury in neonatal mice and rats (25–27).

In contrast to IL-1β, the role of IL-1α in the developing lung is relatively under-studied. However, available evidence supports that the developing lung is uniquely susceptible to the effect of exposure to exogenous IL-1α, and that this effect is distinct from IL-1β in developing lung. Exposing fetal sheep to intra-amniotic (IA) IL-1α during the saccular stage of lung development induces greater inflammatory cell infiltrate compared to either IL-1β or LPS and disrupts normal lung development, but also leads to increased surfactant production and lung compliance (21, 28). However, whether the developing lung response to innate immune challenge by upregulating IL-1α is unknown. However, if pulmonary IL-1α expression in response to innate immune challenge is developmentally regulated, this would necessitate further interrogation of the potentially beneficial, and detrimental effects, of blocking its activity through the use of IL1R antagonism. It is critical to determine whether this distinct role of IL-1α is relevant, as efforts to prevent IL-1R signaling in order to inhibit the detrimental effect of IL-1β will also attenuate the effect of IL-1α (29, 30). Determination of whether the developing lung responds to innate immune challenge with IL-1α upregulation and the transcriptional mechanisms underlying this upregulation if present, is an important first step in answering these questions.

The transcription factor NFκB has been termed the “master regulator the inflammatory response” (31–33). Importantly, in response to (intraperitoneal) IP LPS challenge, its pulmonary activity is developmentally regulated (34). Of note, previous studies have demonstrated that IL-1α is an NFκB target gene (35). Thus, we sought to determine whether endotoxemia induces developmentally-regulated pulmonary IL-1α expression, and if so, whether this occurs via an NFκB dependent mechanism. Identifying IL-1α as a developmentally regulated gene would provide mechanistic insights into the developing innate immune response and provide new therapeutic targets for attenuating lung injury and abnormal development resulting from inflammatory stress.

## Materials and Methods

### Fetal Endotoxemia

C57BL/6 timed pregnant mice were purchased from Charles River Laboratories. Fetal endotoxemia was induced as previously described (36) using a validated model of intrauterine inflammation which results in 100% preterm birth and no maternal mortality. All dams injected at e15 went into preterm labor within 16 h of intrauterine LPS injection, while all dams injected at e19 went into preterm labor within 6 h of the injection. For gene expression studies, tissues were collected from the 4 pups adjacent to the injection site of each uterine horn (37).

### Postnatal Endotoxemia

Adult and newborn (P0) ICR (WT, Taconic) mice were exposed to lethal (50 mg/kg, defined as >90% mortality by 48 h) LPS (O111:B4, L2630, Sigma-Aldrich) challenge. Additionally, newborn (P0), P7 (early alveolar), P28 (late alveolar), and adult ICR (WT) mice were exposed to sublethal (5 mg/kg, defined as >90% survival at 72 h) LPS challenge by intraperitoneal (IP) injection. To interrogate the role of NFκB signaling in the pulmonary response to endotoxemia, newborn (P0) IκBβ^−/−^ mice (kind gift of Dr. Sankar Ghosh) and their WT controls (C57BL/6) were exposed to sublethal (5 mg/kg, defined as >90% survival at 72 h) LPS challenge by intraperitoneal (IP) injection. Following exposure, mice were sacrificed, normal saline was perfused through the right ventricle, and tissue samples were collected and processed as described below. Male mice were used for all experiments. All procedures were approved by the Institutional Animal Care and Use Committee at the University of Colorado (Aurora, CO, USA).

### Tissue Whole Cell Lysate

Lung, liver, spleen, and kidney tissue was homogenized in T-PER (ThermoFisher Scientific) lysis buffer with protease/phosphatase inhibitors (HALT, ThermoFisher Scientific) using the Bullet Blender (Liver: 0.5 mm zirconium oxide beads; Speed 8, 3 min; Lung: stainless steel beads, Speed 10, 4 min). Protein content of supernatant was determined by Bradford assay.

### Cytosolic and Nuclear Extraction

Cytosolic and nuclear extracts were collected using the NE-PER kit (Pierce) according to the manufacturer's instructions, with some modifications. Specifically, following collection of the cytosolic extract, the nuclear pellet was washed and resuspended in cytosolic extraction reagent to completely remove the remaining cytosolic fraction.

### Tissue mRNA Isolation and cDNA Synthesis

For tissue, 30 mg was homogenized in RLT buffer in a Bullet Blender (0.5 mm glass beads; Speed 7, 5 min; NextAdvance), supernatants were collected, and RNA was extracted using the RNeasy Mini Kit (Qiagen) according to the manufacturer's instructions. RNA was assessed for purity and concentration using the NanoDrop (ThermoFisher Scientific), and cDNA synthesized using the Verso cDNA synthesis Kit (ThermoFisher Scientific).

### Cell Culture, LPS Exposure, Pharmacological NFκB Inhibition

RAW 264.7 murine macrophages (ATCC) were cultured according to the manufacturer's instructions. BMDMs were collected from 6 to 10-week-old male C57BL/6 and IκBβ^−/−^ mice and were cultured as previously described (38). Cells were exposed to LPS (RAW cells 1 μg/ml; BMDM 1 μg/ml; O111:B4, L4391, Sigma-Aldrich). To pharmacologically inhibit NFκB activity, cells were exposed to BAY 11-7085 (0–20 μM, Sigma-Aldrich) for 1 h prior to LPS exposure; inhibitors were maintained in the culture media throughout the LPS exposure (39).

### Cellular Protein and mRNA Collection

Whole cell lysates were collected from cultured cells, and protein concentration determined as previously described (40). RNA extraction and cDNA synthesis were performed as previously described (39).

### Immunoblot Analysis

Lysates, cytosolic and nuclear extracts were electrophoresed on a 4-12% polyacrylamide gel (Invitrogen) and proteins were transferred to an Immobilon membrane (Millipore). Membranes were blotted with anti-IκBα (Cell Signaling, #4814), anti-IκBβ (R&D systems, AF5225), anti-p50 (Abcam, ab7971), anti-p65 (Cell Signaling, #8242), anti-IL-1α (R&D systems, AF-400-NA), anti-HDAC (for nuclear extracts, Cell Signaling, #5356), anti-Calnexin (for lysate, Enzo Life Sciences ADI-SPA-860), and anti-GAPDH (for cytosolic extracts, Cell Signaling, #5174). Densitometric analysis was performed using Image Studio (LiCor).

### Analysis of Relative mRNA Levels by RT-qPCR

Relative mRNA levels were evaluated by quantitative real-time PCR using the TaqMan gene expression system (Applied Biosystems). Gene expression of IL-1α (Mm00439620_m1) was assessed with predesigned exon-spanning primers using the StepOnePlus Real Time PCR System (Applied Biosystems). Relative quantitation was performed via normalization to the endogenous control 18S using the cycle threshold (ΔΔCt) method.

### Transfection With WT and Dominant Negative IκBα Overexpression Plasmids

To establish IκB overexpression, RAW 264.7 cells were grown to 70% confluence and transfected with pCMV-IκBα vector (Clontech) or the dominant negative IκBα vector pCMV-IkBaM (Clontech) which has serine to alanine mutations at residues 32 and 36, preventing its phosphorylation and degradation. Cell were transfected using Lipofectamine 2000 (Invitrogen) according to the manufacturer's instructions.

### Chromatin Immunoprecipitation (ChIP)

For each ChIP experiment one set (right and left) of neonatal lungs was used. Chromatin was prepared using the Magna ChIP G tissue kit (Millipore) per protocol with the notable modification of sonicating tissues in nuclear lysis buffer (Millipore). Sonication was performed using the Diagenode Bioruptor Plus for three sets of ten 30 s cycles. DNA was quantified using a spectrophotometer. Twenty-five microgram of chromatin was diluted to a total volume of 500 μL in dilution buffer. Antibodies used for immunoprecipitation included Rabbit IgG (Millipore), anti-p65 (Abcam, Ab7970), and anti-p50 (Cell Signaling, D4P4D). Immunoprecipitations were incubated at 4°C overnight. Enrichment of the IL-1α promoter was assessed by real-time qPCR using SYBR green reagent (Qiagen) and a predesigned IL-1α promoter spanning primer (Qiagen EpiTect ChIP qPCR primer, NM_ 010554.4 (-1)01Kb). Results were expressed as percent input.

### Immunohistochemistry

Neonatal lungs sections were collected and fixed with 4% PFA and stored. Samples were then heated to deparaffinize and rehydrated with xylene and ethanol.

For DAB immunohistochemistry slides were quenched with BLOXALL (Vector labs) and blocked. They were then incubated with IL-1α primary antibodies (R&D systems, AF-400-NA), followed by a biotinylated secondary antibody, VECTASTATIN. R.T.U. ABC Reagent, and finally stained with DAB peroxidase (Vector Labs). They were counterstained with methyl green. IL-1α staining was visualized using the Olympus IX83 microscope and Olympus DP80 camera at Å~40 magnification using Olympus CellSens software.

For immunofluorescence, antigen retrieval was performed with citric acid, tissues were permeabilized with 0.5% triton, quenched with glycine, and blocked. They were incubated with IL-1α (R&D systems, AF-400-NA) and pro-surfactant protein C (Millipore, AB3786) primary antibodies overnight followed by incubation with secondary antibodies (Alexa fluor anti-rabbit donkey 647 and anti-goat donkey 594) for 1 h. Staining was visualized using the Olympus IX83 microscope and Olympus DP80 camera using Olympus CellSens software.

### Statistical Analysis

For comparison between treatment groups, the null hypothesis that no difference existed between treatment means was tested by Student's *t*-test for two groups and two-way ANOVA for multiple groups with potentially interacting variables (genotype, LPS exposure), with statistical significance between and within groups determined by means of Bonferroni method of multiple comparisons (Prism, GraphPad Software, Inc.). Statistical significance was defined as *p* < 0.05.

## Results

### Endotoxemia Induces Pulmonary IL-1α mRNA Expression During the Saccular and Alveolar Stages of Lung Development

**Figure 1:**
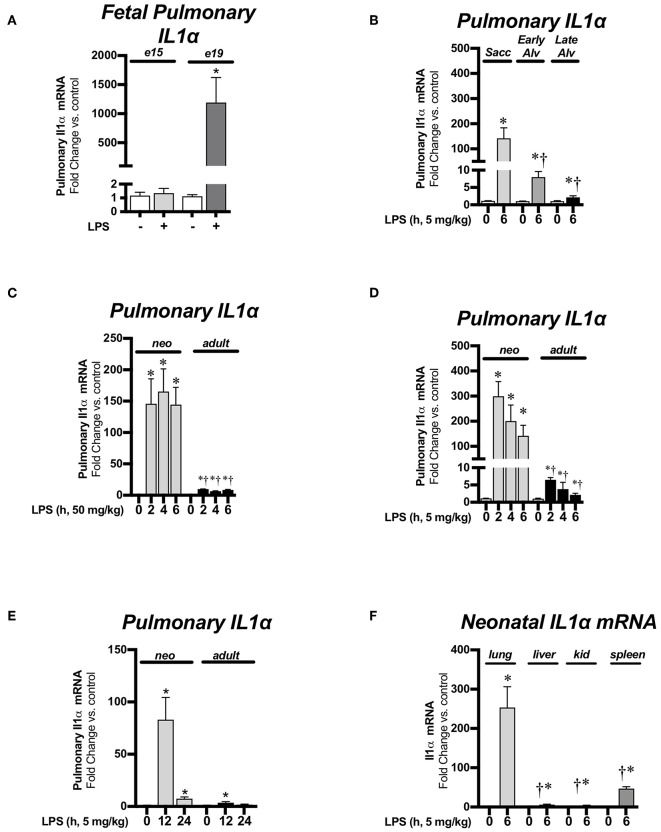
Endotoxemia induces pulmonary IL-1α mRNA expression during the saccular and alveolar stages of lung development. Fold-increases in pulmonary IL-1α mRNA expression **(A)** after intrauterine LPS (250 μg) injection during the pseudoglandular/cannalicular (e15) and saccular (e19) stages of fetal lung development. Data expressed as mean ± SEM (*n* = 9–12 per time point). ^*^p < 0.05 vs. unexposed control. **(B)** After sublethal (5 mg/kg, IP; 6 h) LPS exposure during the saccular (P0), early alveolar (P7), and late (adult) stages of postnatal alveolar lung development. Data expressed as mean ± SEM (*n* = 4–25 per time point). ^*^p < 0.05 vs. unexposed control. *^†^p* < 0.05 vs. time-matched LPS-exposed P0 lung. **(C)** After lethal LPS exposure (50 mg/kg, IP, 0–6 h) in neonates (P0) and adults. Data expressed as mean ± SEM (*n* = 4–9 per time point) ^*^p < 0.05 vs. unexposed control. *^†^p* < 0.05 vs. time-matched LPS-exposed adult lung or **(D)** after sublethal LPS exposure (5 mg/kg, IP, 0–6 h) in neonates (P0) and adults. Data expressed as mean ± SEM, (*n* = 4–25 per time point) ^*^p < 0.05 vs. unexposed control. *^†^p* < 0.05 vs. time-matched LPS-exposed adult lung and **(E)** after sublethal LPS injections (5 mg/kg, 12–24 h) in neonates (P0) and adults. Data expressed as mean ± SEM (*n* = 3–11 per time point) ^*^p < 0.05 vs. unexposed control. **(F)** Fold-increases in pulmonary, liver, kidney, and spleen IL-1α mRNA expression 6 h after sublethal LPS exposure (5 mg/kg, IP, 6 h). Data expressed as mean ± SEM (*n* = 5–9 per time point). ^*^p < 0.05 vs. unexposed control tissue, *^†^p* < 0.05 vs. time-matched LPS-exposed lung.

For this study, we compared LPS-induced pulmonary IL-1α expression during the pseudoglandular/canalicular (e15), saccular (e19 and P0), early alveolar (P7), late alveolar (P28), and adult stages of lung development. There was no significant change in pulmonary IL-1α expression in endotoxemia-exposed pseudoglandular/canalicular (e15) lung (Figure 1A). In contrast, there was robust IL-1α expression in saccular lung (e19, Figure 1A; p0, Figure 1B). Of note, the level of endotoxemia-induced IL-1α induction attenuated as lung development progressed past the saccular stage (P0) to the early (P7) and late (P28) alveolar stage (Figure 1B). Next, we evaluated pulmonary IL-1α expression over a time course in neonatal (P0) and adult mice exposed to lethal (50 mg/kg, Figure 1C) or sublethal (5 mg/kg, Figure 1D) endotoxemia. Neonatal pulmonary IL-1α expression was significantly increased compared to adults in response to both sublethal and lethal endotoxemia (Figures 1C,D). We then evaluated sustained IL-1α expression in mice exposed to sublethal endotoxemia. Of note, neonatal mice exposed to lethal endotoxemia did not survive past 12–24, making evaluation of IL-1α at these later time points impossible. Importantly, IL-1α expression remained significantly elevated in the neonatal lung 24 h following the one-time exposure to sublethal endotoxemia on the day of birth while IL-1α expression was not significantly elevated at 24 h in the lungs of adult mice (Figure 1E). Compared to other organs tested, including the liver, kidney and spleen, endotoxemia-induced IL-1α expression was greatest neonatal lung (Figure 1F).

### Endotoxemia Induces Pulmonary IL-1α Protein Expression During the Saccular Stage of Lung Development

**Figure 2:**
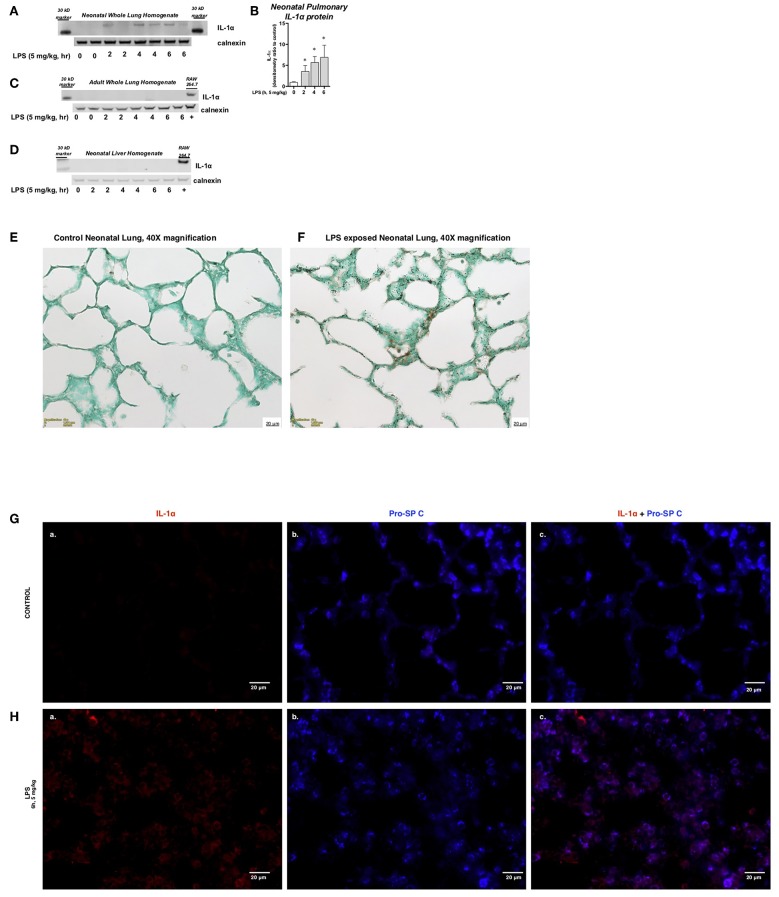
Endotoxemia induces pulmonary IL-1α protein expression during the saccular stage of lung development. **(A)** Representative Western blot of neonatal pulmonary IL-1α protein expression LPS exposure (IP, 5 mg/kg, IP, 2–6 h) with calnexin used as the loading control. Each time point has sample from 2 different mice. **(B)** Densitometric evaluation of neonatal pulmonary IL-1α expression. For densitometric evaluation, results are normalized to GAPDH, and expressed as a ratio to unexposed control. Data expresses as mean ± SEM (*n* = 4 per time point). ^*^p < 0.05 vs. unexposed control. Representative Western blot of **(C)** adult pulmonary and **(D)** neonatal liver IL-1α protein expression LPS exposure (IP, 5 mg/kg, IP, 2–6 h) with calnexin used as the loading control. Each time point has sample from 2 different mice. **(E,F)** Representative DAB staining of **(E)** control and **(F)** LPS-exposed (5 mg/kg, IP, 4 h) neonatal lung. Internal scale bar 20 μM. Imaged at 40x. **(G,H)** Representative immunofluorescence staining of **(G)** control and **(H)** LPS-exposed (5 mg/kg, IP, 6 h) neonatal lung. IL-1α was stained in red (a) and Type II epithelial cells [Pro-SP C; (b)] stained in blue. Image (c) is overlay of (a,b). Internal scale bar 20 μM. Imaged at 60x.

We next assessed whether the observed transcriptional response was associated with measurable changes in pulmonary IL-1α protein expression. Western blot analysis confirmed the presence of IL-1α protein in whole cell lysate obtained from the lungs of endotoxemic neonatal mice (Figures 2A,B). In contrast, no IL-1α protein was detected in the whole cell lystate obtained from the lungs of endotoxemic adult mice (Figure 2C) or the liver of endotoxemic neonatal mice (Figure 2D). Immunohistochemistry confirmed the presence of IL-1α protein in the lungs of LPS-exposed neonatal mice (Figures 2E,F). Having noted the presence of IL-1α protein by immunohistochemistry, we used immunofluorescence to determine the cellular source of IL-1α in LPS-exposed neonatal mice. We were unable to detect IL-1α in the lungs of neonatal mice prior to LPS exposure (Figures 2Ga–c) and found that IL-1α co-stained strongly with SPC following LPS exposure (Figures 2Ha–c). Together, these results demonstrate that the increase in pulmonary lL-1α mRNA expression in the lungs of neonatal mice is associated with increased protein expression in Type II epithelial cells.

### Endotoxemia Induces Sustained Pulmonary NFκB Activation in Neonatal Mice

**Figure 3:**
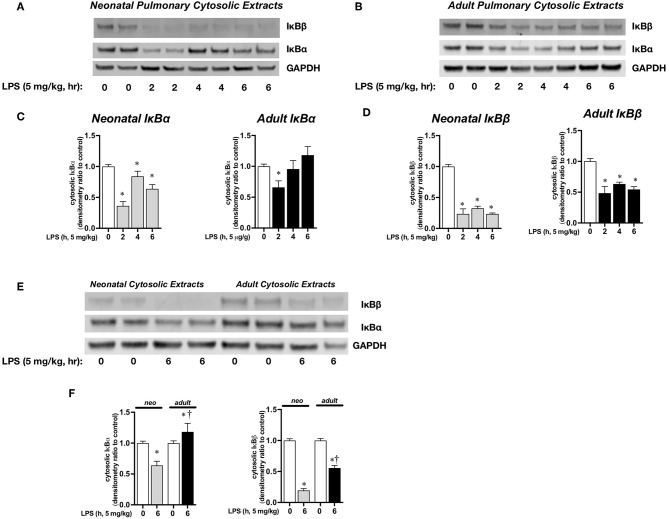
Endotoxemia induces cytosolic degradation of the NFκB inhibitory proteins IκBα and IκBβ. Representative Western blots of IκBα and IκBβ in **(A)** neonatal and **(B)** adult pulmonary cytosolic extracts after LPS exposure (IP, 5 mg/kg, 0–6 h). Each time point has sample from 2 different mice. GAPDH as loading control. Densitometric analysis of **(C)** IκBα and **(D)** IκBβ in adult and neonatal pulmonary cytosolic extracts. For densitometric evaluation, results are normalized to GAPDH, and expressed as a ratio to unexposed control. Data expressed as mean ± SEM (*n* = 3–9 per time point). ^*^p < 0.05 vs. unexposed control. **(E)** Representative Western blot of IκBα and IκBβ in adult and neonatal pulmonary cytosolic extracts after LPS exposure (5 mg/kg, IP, 6 h). Each time point has sample from 2 different mice. **(F)** Denistometric analysis of IκBα and IκBβ in adult and neonatal pulmonary cytosolic extracts. For densitometric evaluation, results are normalized to GAPDH and expressed as a ratio to unexposed control. Data expressed as mean ± SEM (*n* = 3–9 per time point). ^*^p < 0.05 vs. unexposed control. *^†^P* < 0.05 vs. time-matched P0.

We next sought to determine the transcriptional mechanisms underlying increased IL-1α expression in neonatal mice. Previous studies have demonstrated a role played by the transcription factor NFκB in IL-1α expression (35). First, we assessed pulmonary cytosolic extracts from LPS-exposed adult and neonatal mice for evidence of degradation of the NFκB inhibitory proteins IκBα and IκBβ. In LPS-exposed neonatal mice, IκBα degraded within 2 h of exposure, and remained significantly lower compared to baseline at 6 h of exposure (Figures 3A,C). In contrast, in LPS-exposed adult mice, IκBα degraded within 2 h of exposure, and returned to baseline by 6 h of exposure (Figures 3B,C). IκBβ levels remained lower than baseline throughout the exposure in both neonatal and adult mice (Figures 3A,B,D). However, when compared directly, cytosolic levels of IκBβ remained significantly lower in the neonatal compared to adult lung (Figures 3E,F). This finding is critically important, as the re-accumulation of cytosolic IκBβ is marks termination of NFκB activity (41). Based on these findings, we used 1 h of exposure as a marker of early NFκB activity, and 5 h as a marker of late NFκB activity in the next set of experiments.

**Figure 4:**
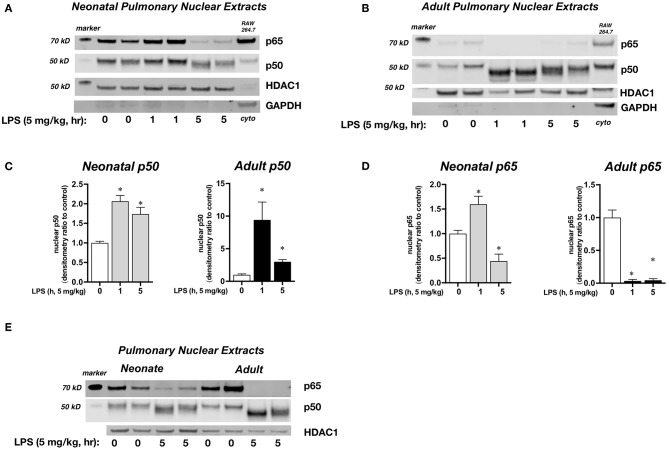
Effect of endotoxemia on the nuclear translocation of the NFκB subunits p65 and p50. **(A,B)** Representative Western blots of p65 and p50 in **(A)** neonatal and **(B)** adult pulmonary nuclear extracts after LPS exposure (IP, 5 mg/kg, 0, 1, and 5 h). Each time point has sample from 2 different mice. HDAC1 as loading control, with GAPDH shown as evidence of purity of nuclear samples. Densitometric analysis of **(C)** p50 and **(D)** p65 in adult and neonatal pulmonary nuclear extracts. For densitometric evaluation, results are normalized to HDAC1, and expressed as a ratio to unexposed control. Data expressed as mean ± SEM (*n* = 4–5 per time point). ^*^p < 0.05 vs. unexposed control. **(E)** Representative Western blot of p50 and p65 in neonatal and adult pulmonary nuclear extracts after LPS exposure (5 mg/kg, IP, 0, 1 and 5 h). Each time point has sample from 2 different mice. HDAC1 as loading control.

We next sought to confirm that cytosolic NFκB inhibitory protein degradation was associated with pulmonary nuclear translocation of the NFκB subunits p65 and p50. For this set of experiments, we used 1 h exposure to evaluate the early pulmonary nuclear translocation of NFκB subunits, and a 5 h exposure to evaluate later events. In LPS-exposed neonatal mice, there was significant pulmonary nuclear accumulation of p50 at 1 and 5 h, and significant accumulation of p65 at 1 h and persistence of nuclear p65 at 5 h (Figures 4A,C,D). In contrast, in LPS-exposed adult mice, there was robust nuclear accumulation of p50 while levels of p65 decreased and were near absent at 1 and 5 h of exposure to sublethal endotoxemia (Figures 4B–D). The difference between neonatal and adult mice in the composition of pulmonary nuclear NFκB subunits following LPS exposure becomes clear when compared directly (Figure 4E). By 5 h of exposure, p65 remains in the nuclear extracts of pulmonary tissue of LPS-exposed neonatal mice, while little to none can be detected in the nuclear extracts of pulmonary tissue of LPS-exposed adult mice (Figure 4E). Thus, at later time points following LPS exposure, only the neonatal lung demonstrates nuclear p50 and p65. This observation is critically important, as the activity of inhibitory proteins and the nuclear translocation of NFκB dimers (e.g., dimers containing only p50 vs. dimers composed of p65 and p50) determine the selectivity of the NFκB transcriptome (42).

### The NFκB Subunits p65 and p50 Bind to the Neonatal Pulmonary IL-1α Promoter in Endotoxemia

**Figure 5:**
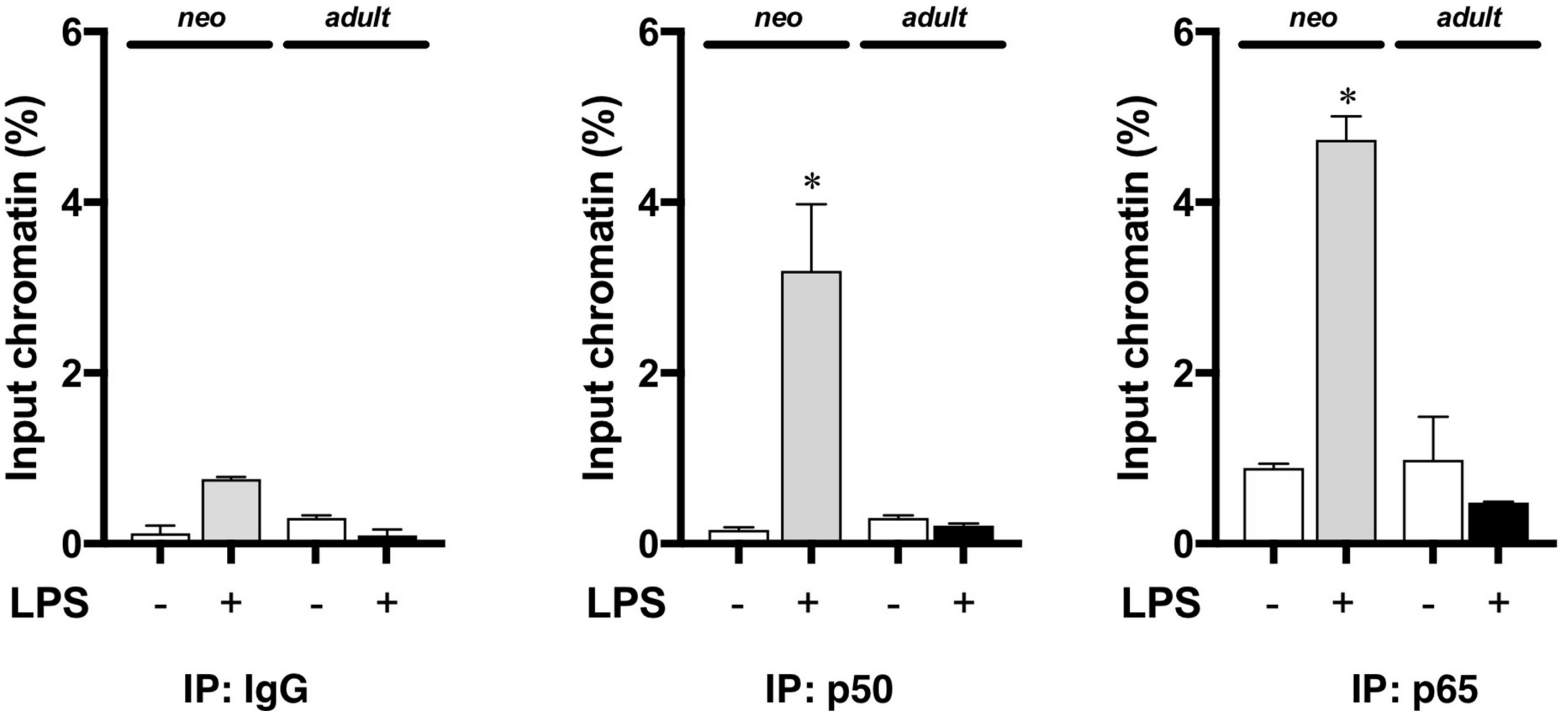
The NFκB subunits p50 and p65 are bound to the IL-1α promoter the lungs of endotoxemic neonatal mice. Neonatal and adult mice were exposed to LPS (5 mg/kg, IP, 5 h), and whole lung homogenates subjected to chromatin immunoprecipitation with anti-p65 and anti-p50 and analyzed by qPCR targeting the IL-1α promoter. Results show average percent input of RT qPCR for amplification of the IL-1α promoter run in triplicate. IgG was used as a negative control. Data expressed as mean ± SEM. Results are representative of 3 separate experiments. ^*^p < 0.05 vs. unexposed WT control.

Having demonstrated that nuclear NFκB translocation in the neonatal lung was temporally related to LPS-induced IL-1α expression, we next evaluated whether NFκB was directly implicated in IL-1α expression. Thus, we performed chromatin immunoprecipitation of p50 and p65 interrogating the IL-1α promoter. We found that with endotoxemia, both p50 and p65 were present at the IL-1α promoter in the neonatal lung at 5 h of exposure (Figure 5). In contrast, we did not detect the NFκB subunits p50 or p65 at the IL-1α promoter in the lungs of endotoxemic adult mice (Figure 5). These results demonstrate that developmentally regulated expression of LPS-induced pulmonary IL-1α expression is NFκB dependent.

### LPS Induces NFκB-Mediated IL1α Expression in Macrophages

**Figure 6:**
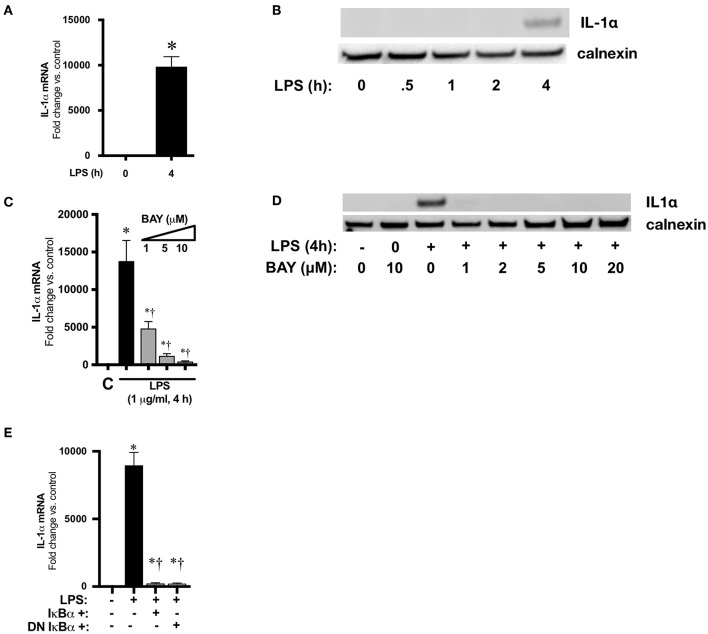
NFκB mediates LPS-induced IL-1α expression in RAW 264.7 macrophages. **(A)** Fold-increase in IL-1α mRNA expression after LPS exposure (1 μg/ml, 4 h). Data expressed as mean ± SEM (*n* = 4 per time point). ^*^p < 0.05 vs. unexposed control. **(B)** Representative Western blot showing IL-1α protein expression in whole cell lystates obtained from LPS exposed (1 μg/ml, 0.5–4 h) RAW 264.7 macrophages. Calnexin as loading control. **(C)** Fold-increase in gene expression of IL-1α in RAW 264.7 macrophages following LPS exposure (4 h, 1 μg/ml) or BAY 11-7085 pretreatment (1 h, 1–10 μM) and LPS exposure. Data expressed as mean ± SEM (*n* = 4 per timepoint). ^*^p < 0.05 vs. control, *^†^p* < 0.05 vs. LPS-exposed. **(D)** Representative Western Blot of IL-1α protein expression in whole cell lystates obtained from RAW 264.7 macrophages 4 h after LPS exposure alone or LPS after pretreatment (1 h) with BAY 11-7085 (1–20 μM). Calnexin as loading control. **(E)** Fold-increase in IL-1α mRNA expression following LPS exposure (1 μg/ml, 5 h) or wild-type (WT) or dominant negative (DN) IκBα overexpression and LPS exposure. Data expressed as mean ± SEM (*n* = 4 per time point). ^*^p < 0.05 vs. control, *^†^p* < 0.05 vs. LPS-exposed.

We next used an *in vitro* cell culture model to elucidate the transcriptional regulation of IL-1α following LPS exposure. Similar to the neonatal lung, LPS induces the nuclear translocation of the NFκB subunits p50 and p65 in immortalized murine macrophages (RAW 264.7) (43). Furthermore, following exposure to LPS, RAW 264.7 macrophages displayed marked induction of IL-1α mRNA (Figure 6A) and protein (Figure 6B) expression. Having identified RAW 264.7 as a source of LPS-induced IL-1α expression, we next sought to link LPS-induced NFκB signaling to IL-1α expression. Pre-treatment with the pharmacologic NFκB inhibitor BAY 11-7085 inhibited LPS-induced IL-1α mRNA (Figure 6C) and protein (Figure 6D) expression in a dose dependent manner.

To exclude off-target effects of BAY 11-7085 and more directly implicate NFκB signaling in LPS-induced IL-1α expression, RAW 264.7 cells were transfected with plasmids overexpressing wild-type (WT) and dominant negative (DN) IκBα. The dominant negative IκBα plasmid results in expression of IκBα in which serine 32/36 have been mutated to phenylalanine, preventing phosphorylation and subsequent degradation. Both WT and DN IκBα overexpression significantly attenuated LPS-induced IL-1α expression (Figure 6E). These results implicate LPS-induced NFκB activation in the transcriptional regulation of IL-1α.

### IκBβ^−/−^ Bone Marrow Derived Macrophages (BMDM) Have Abbreviated LPS-Induced NFκB Activation and Attenuated IL-1α Expression

**Figure 7:**
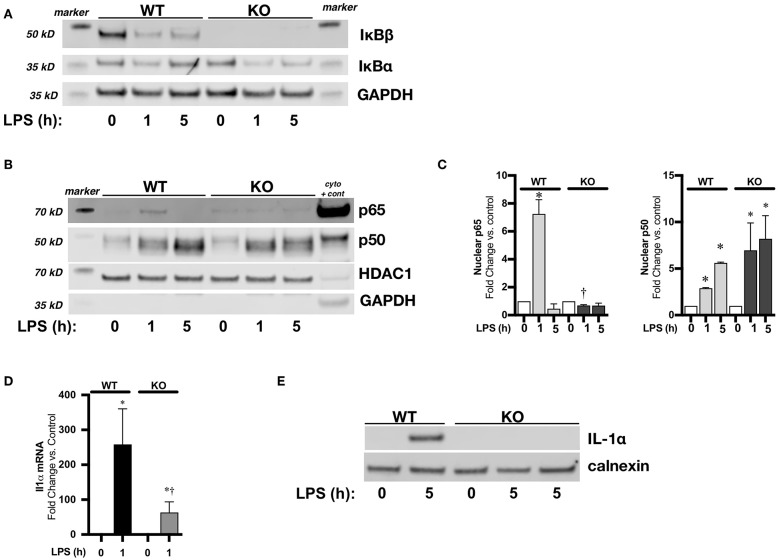
IκBβ^−/−^ bone marrow derived macrophages (BMDM) have abbreviated LPS-induced NFκB activation and attenuated IL-1α expression. **(A)** Representative Western blots of IκBα and IκBβ in cytosolic fractions in LPS exposed (1 μg/ml, 1–5 h) WT and IκBβ^−/−^ BMDM with GAPDH used as a loading control. **(B)** Representative Western blot of p65 and p50 in nuclear fractions in LPS exposed (1 μg/ml, 1–5 h) WT and IκBβ^−/−^ BMDM with HDAC used as a loading control. A cytosolic sample was run on the same blot to serve as a positive control, and labeled as such (cyto + cont). GAPDH shown as evidence of purity of nuclear samples **(C)** Denistometric analysis of p65 and p50 in WT and IκBβ^−/−^ BMDM nuclear extracts. For densitometric evaluation, results are normalized to HDAC1 and expressed as a ratio to unexposed control. Data expressed as mean ± SEM (*n* = 3 per time point). ^*^p < 0.05 vs. unexposed genotype control; *^†^P* < 0.05 vs. time-matched WT LPS-exposed. **(D)** Fold-increase in IL-1α mRNA expression in WT and IκBβ^−/−^ BMDM following LPS exposure (1 μg/ml, 1 h) Data expressed as mean ± SEM (*n* = 6 per timepoint). ^*^p < 0.05 vs. control, *^†^p* < 0.05 vs. time-matched WT LPS-exposed. **(E)** Representative Western blot of IL-1α in whole cell lysates obtained from LPS exposed (1 μg/ml, 5 h) WT and IκBβ^−/−^ BMDM. IκBβ^−/−^ BMDM exposed to 5 h of LPS has a sample from 2 different exposures. Calnexin used as a loading control.

Use of pharmacologic inhibitors and transient transfection can have unanticipated off-target effects. It is well-established that the nuclear action of IκBβ stabilizes NFκB/DNA binding leading to the sustained expression of select pro-inflammatory genes (44, 45). Thus, to further establish a relationship between IκBβ/NFκB signaling and LPS-induced IL-1α expression, we performed experiments using BMDM isolated from IκBβ^−/−^ mice. We found that following exposure to LPS, cytosolic IκBβ levels in WT BMDM decreased, consistent with LPS-induced IκBβ/ NFκB signaling (Figure 7A). As expected, IκBβ^−/−^ BMDM did not express IκBβ (Figure 7A). Next, we compared LPS-induced NFκB nuclear translocation in LPS exposed WT and IκBβ^−/−^ BMDM. At the 1-h time point, both p65 and p50 were significantly elevated in LPS-exposed WT BMDM (Figures 7B,C). In LPS-exposed IκBβ^−/−^ BMDM, nuclear levels of p65 were significantly lower than similarly exposed WT BMDM (Figures 7B,C). Nuclear translocation of p65 and p50 was associated with IL-1α mRNA and protein expression in LPS-exposed WT BMDM (Figures 7D,E). Both IL1α mRNA (Figure 7D) and protein (Figure 7E) were significantly decreased in IκBβ^−/−^ compared to WT BMDM. These results corroborate a role played by IκBβ/NFκB signaling, and a transcriptional role played by both p65 and p50 in LPS-induced IL-1α expression.

### Neonatal IκBβ^−/−^ Mice Have Attenuated Endotoxemia-Induced Pulmonary IL-1α Expression

**Figure 8:**
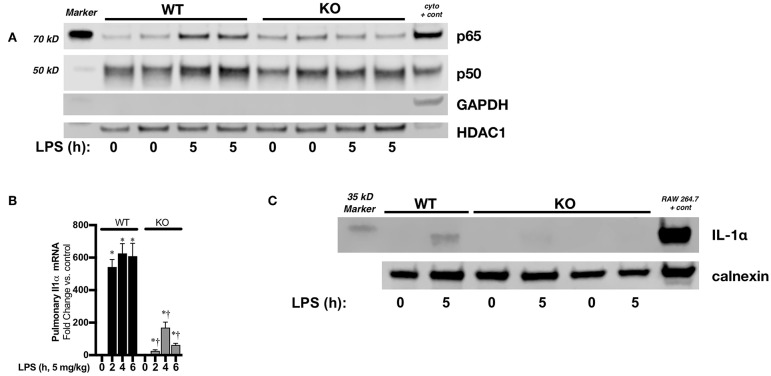
Neonatal IκBβ^−/−^ mice have attenuated LPS-induced pulmonary IL-1α expression. **(A)** Representative Western blot of p65 and p50 in WT and IκBβ^−/−^ pulmonary nuclear extracts after LPS exposure (5 mg/kg, IP, 5 h). Each time point has sample from 2 different mice. A cytosolic sample was run on the same blot to serve as a positive control, and labeled as such (cyto + cont). **(B)** Fold-increase in pulmonary IL-1α mRNA expression in WT and IκBβ^−/−^ neonatal mice following LPS exposure (5 mg/kg, 0–6 h) Data expressed as mean ± SEM (*n* = 6 per timepoint). ^*^p < 0.05 vs. control, *^†^p* < 0.05 vs. time-matched WT LPS-exposed. **(C)** Representative Western blot of IL-1α in pulmonary whole cell lysates from LPS exposed (5 mg/kg, 5 h) WT and IκBβ^−/−^ neonatal mice with calnexin used as a loading control.

Next, we sought to confirm a relationship between sustained pulmonary LPS-induced NFκB signaling and neonatal IL-1α expression *in vivo*. In endotoxemic neonatal IκBβ^−/−^ mice, both p50 and p65 have returned to baseline levels by 5 h of exposure (Figure 8A). Accordingly, pulmonary IL-1α mRNA (Figure 8B) and protein (Figure 8C) were significantly decreased in LPS exposed IκBβ^−/−^ neonatal mice. These results corroborate the role played by sustained LPS-induced IκBβ/NFκB signaling and nuclear p65 and p50 in pulmonary IL-1α expression in endotoxemic neonatal mice.

## Discussion

We have demonstrated that sustained pulmonary NFκB signaling leads to developmentally regulated pulmonary IL-1α expression in neonatal endotoxemia. Our data show that IL-1α expression is robustly and uniquely upregulated during the saccular stage of pulmonary development in response to endotoxemia. In contrast, IL-1α is not upregulated in the preceding pseudoglandular/cannalicular stage and decreases sharply as the lung progresses from the saccular to alveolar to adult stages of development. Importantly, we demonstrate that this developmentally regulated expression is NFκB dependent. Firstly, the kinetics of LPS-induced NFκB activation are significantly different between neonatal and adult mice. Notably, pulmonary p65/p50 signaling is sustained after LPS exposure in neonatal mice. Furthermore, ChIP demonstrated presence of both p65 and p50 at the IL-1α promoter in the lungs of LPS exposed neonatal mice. Finally, in genetically modified IκBβ^−/−^ BMDMs and neonatal mice with abbreviated LPS-induced NFκB activation, IL-1α expression was significantly attenuated. Together, these results demonstrate that the developing lung is subject to unique innate immune signaling and target gene expression following an inflammatory challenge. These findings have implications for therapeutic approaches to attenuate lung injury and subsequent abnormal lung development following exposure to perinatal inflammatory stress. Further work must be done to determine whether these developmentally regulated pathways and target genes mediate any potential beneficial effects, such as increased surfactant production, in the setting of inflammatory stress, prior to clinical attempts to broadly inhibit their activity.

Perinatal inflammatory injuries such as chorioamnionitis and sepsis contribute to the pathogenesis of BPD (4–6). The transcription factor NFκB has been termed the “master regulator the inflammatory response,” and inhibiting its activity has been actively pursued as a way to prevent injury associated with inflammation (41, 46, 47). However, pre-clinical studies show that *complete NF*κ*B inhibition* disrupts normal lung development (48, 49). and exacerbates neonatal lung injury induced by endotoxemia (34, 50). Maturational differences in NFκB activation also exist, as neonatal T-lymphocytes and polymorphonuclear lymphocytes show increased NFκB activation in response to various stimuli when compared to their adult counterparts (51, 52). Similarly, fetal lung fibroblasts, in contrast to adult cells, demonstrate hyperoxia-induced NFκB activation (53). *In vivo*, hyperoxia-induced NFκB activation is enhanced in alveolar epithelium and endothelium of neonatal mice compared to that of adults (54). Therefore, a better understanding of the mechanisms underlying differences between inflammatory stress induced NFκB activation in the neonate and adult could reveal therapeutic targets to attenuate lung injury.

Our results demonstrate that there are fundamental differences between neonatal and adult mice in the degree and duration of LPS-induced pulmonary NFκB activation. Furthermore, we add mechanistic data that links developmentally regulated LPS-induced NFκB activation to pulmonary IL-1α expression. Specifically, we demonstrate the sustained nuclear presence of the NFκB subunits p65 and p50 in the neonatal mouse lung after LPS exposure. Previous studies have shown that sustained NFκB nuclear signaling mediates the inducible expression of multiple pro-inflammatory genes (37, 39, 43, 55), including IL-1α (44, 45). Through the study of IκBβ^−/−^ mice, we have demonstrated that LPS-induced pulmonary NFκB activity can be abbreviated in neonatal mice with resulting attenuated IL-1α. However, other mechanisms underlying differential NFκB activity may explain the differences seen between adult and neonatal mice. Of note, much work has been done on the mechanisms underlying termination of NFκB signaling via p65 degradation (56–60). Whether these mechanisms are at play in the adult lung, and whether they are attenuated in the neonatal lung is unknown.

The role of IL-1β in promoting lung injury and abnormal development after inflammatory insults has been relatively well-characterized in both clinical and pre-clinical studies (7, 9, 21–27, 61). Additionally, pretreatment with an IL-1 receptor antagonist can mitigate lung injury induced by LPS or hyperoxia (23, 25, 27). However, the IL-1 receptor (IL1R1) is activated by two distinct genes, IL-1α and IL-1β. Our findings, as well as previous reports, demonstrate that IL-1α may play a unique role in mediating the innate immune response in the developing lung. Fetal sheep exposed to intra-amniotic (IA) IL-1α during the saccular stage of lung maturation mount a greater inflammatory response and have more disrupted lung development than those exposed to IL-1β or LPS (28). However, both fetal sheep and rabbits exposed to intratracheal IL-1α show increased surfactant protein and saturated phosphatidylcholine production as well as improved lung compliance after birth (28, 62, 63). Of note, while IL-1β is expressed in cells of hematopoietic origin, IL-1 α is present in multiple cell types (64). Our results demonstrate that following systemic exposure to LPS, the neonatal lung responds with by upregulating IL-1α expression in the Type II epithelial cell. Thus, in addition to a pro-inflammatory role, IL-1α may play a protective role by accelerating lung maturity and surfactant production. Other studies have demonstrated that IL-1α plays a role in neonatal susceptibility to sepsis (65). Additionally, IL-1α is uniquely upregulated in LPS-exposed neutrophils and monocytes isolated from cord blood when compared to similarly exposed adult cells (66, 67). This report adds to our understanding of the unique transcriptional regulation in the developing lung that underlies inflammatory stress induced IL-1α expression. Furthermore, these findings necessitate consideration of any potentially deleterious effects of using IL-1 receptor antagonists in the perinatal period.

Our findings add to a growing body of pre-clinical (65) and clinical data indicating developmentally regulated innate immune-mediated expression of IL-1α specific to the neonatal period. Furthermore, available data demonstrate that the developing lung is uniquely susceptible to the downstream effects of IL-1α, both in terms of inducing inflammation and enhancing maturation. Whether these potentially injurious and protective effects can be mechanistically dissected is unknown. Of note, IL-1α acts as an alarmin with activity both at the cell surface and in the nucleus. IL-1α can undergo post-translational modifications that create an N terminal piece containing a nuclear localization sequence as well as the C terminal piece responsible for IL-1R1 binding (64). While the role of nuclear IL-1α has not been well-characterized, it suggests a potential role of IL-1α in neonatal lung injury that is independent of IL-1R1 mediated signaling. These mechanisms remain unexplored.

Our study has several limitations. *In vitro*, we have demonstrated that both pharmacologic and genetic inhibition of NFκB activity attenuates LPS-induced IL-1α expression. While these experiments demonstrate mechanistically that NFκB is necessary for LPS-induced IL-1α expression, these cells are not developmentally relevant to the neonatal period. The observation that LPS-induces IL-1α expression in macrophages isolated from adult animals raises the possibility that there are unique inhibitory forces preventing IL-1α expression in the adult lung. These studies need to be performed. Importantly, we have not yet been able to pharmacologically or genetically prevent lung-specific IκBβ/NFκB signaling *in vivo*. Previous studies have demonstrated that LPS-induced NFκB activity is abbreviated in IκBβ^−/−^ mice (44, 45), and we show here that these neonatal mice have attenuated IL-1α expression. However, we did not evaluate the degree of pulmonary injury and subsequent abnormal development in these mice. Importantly, the IκBβ/NFκB signaling also affects the expression of a diverse set of inflammatory genes including IL-1β, IL6, and IL-12β (44, 45), so we would not be able to ascribe any finding specifically to attenuated IL-1α expression. Therefore, any differences in outcomes observed between LPS-exposed neonatal WT and IκBβ^−/−^ mice could not be directly mechanistically linked to differences in IL-1α expression. Appropriate cell-type specific models of impaired IL-1α expression need to be investigated. Regardless, we have uncovered developmentally regulated signaling and target gene expression; the implications of these findings remain to be determined.

We conclude that endotoxemia exposure during the critical stage of saccular lung development induces IL-1α expression via IκBβ/NFκB mediated signaling. Furthermore, we have identified innate immune signaling pathways and target gene expression unique to the developing lung. Our robust analysis supports further study of the implications of targeting IκBβ/NFκB signaling in the developing lung exposed to inflammatory stimuli. Such targeting may prevent inflammatory injury that contributes the development of BPD.

## Data Availability

The datasets generated for this study are available on request to the corresponding author.

## Ethics Statement

All procedures were approved by the Institutional Animal Care and Use Committee at the University of Colorado (Aurora, Colo., USA).

## Author Contributions

BB, SM, and CW conceived and designed the study and prepared figures. BB, RD, LN, SM, and CW performed experiments, analyzed data, and interpreted results of experiments. BB drafted the manuscript. BB, RD, LN, SM, SG, and CW edited and revised manuscript and approved final version of manuscript.

### Conflict of Interest Statement

The authors declare that the research was conducted in the absence of any commercial or financial relationships that could be construed as a potential conflict of interest.
